# Performance of Children With Donnai-Barrow Syndrome After Cochlear Implantation: A Case Report

**DOI:** 10.7759/cureus.21063

**Published:** 2022-01-09

**Authors:** Mohammed Yousef Alyousef, Alanoud A Abuhaimed, Dania M Alkelabi, Maram AlKahtani, Einas M Yousef, Medhat F Yousef

**Affiliations:** 1 Medicine, King Saud University, Riyadh, SAU; 2 Otolaryngology, MED-EL Medical Electronics, Riyadh, SAU; 3 Basic Medical Sciences, College of Medicine, Dar Al Uloom University, Riyadh, SAU; 4 Audiology, King Abdullah Ear Specialist Centre (KAESC) King Saud University Medical City, Riyadh, SAU

**Keywords:** cochlear implantation., lrp2 gene, sensorineural hearing loss, cap and sir, donnai-barrow syndrome

## Abstract

Donnai-Barrow syndrome (DBS) is a rare autosomal recessive hereditary disorder that affects a variety of body systems. One of the most common symptoms in DBS patients is severe bilateral sensorineural hearing loss. The objective of this report is to highlight the performance of such patients after receiving cochlear implants as a management of their hearing loss. We reviewed the medical records of two cousins diagnosed with DBS before and after cochlear implantation, with a particular focus on their auditory and language performance. After receiving the cochlear implant, both patients showed substantial progress in auditory and speech perception, as well as their intelligence quotients, allowing them to join mainstream schools. In conclusion, our findings showed that cochlear implantation can be considered an ideal approach for the management of DBS patients who suffer from bilateral sensorineural hearing loss.

## Introduction

Donnai-Barrow syndrome (DBS)/facio-oculo-acoustico-renal (FOAR) syndrome (MIM# 222448) is an extremely rare disorder that affects multiple body systems and is inherited in an autosomal recessive pattern [1,2]. Few studies assessed its prevalence to be less than one case per 1,000,000 births, with only about 50 cases reported worldwide [3,4]. Many of the affected individuals were born to consanguineous parents [5,6]. DBS/FOAR is caused by a mutation in the low-density lipoprotein receptor-related protein 2 (*LRP2*) gene, which encodes megalin, a multiligand endocytic receptor expressed in many absorptive tissues [6]. Although DBS and FOAR are considered as one entity; the presence of proteinuria, corpus callosum agenesis, and diaphragmatic hernia is associated with FOAR [7].

DBS/FOAR is characterized by specific facial features such as a V-shaped point in the hairline in the middle of the forehead (Widow’s peak), large anterior fontanelle, wide metopic suture, posteriorly rotated ears, down slanted palpebral fissures, hypertelorism, enlarged globes leading to the appearance of prominent eyes, depressed nasal bridge, and short nose with broad or bifid tip [1,8]. Affected individuals potentially develop multi-system abnormalities including bilateral sensorineural hearing loss (SNHL), visual impairment, mild to moderate intellectual disability, developmental delay, absent or underdeveloped corpus callosum, congenital diaphragmatic hernia, and opening in the abdominal wall [9].

Due to the rarity of the disease, information on the auditory performance, speech, and language development of children with DBS are scarce. In this report, we described two cases of DBS with bilateral SNHL who underwent cochlear implantation. The objective of this report is to highlight the performance of those patients after receiving cochlear implants (CI) as a management for their hearing loss.

## Case presentation

Case 1

**Figure 1:**
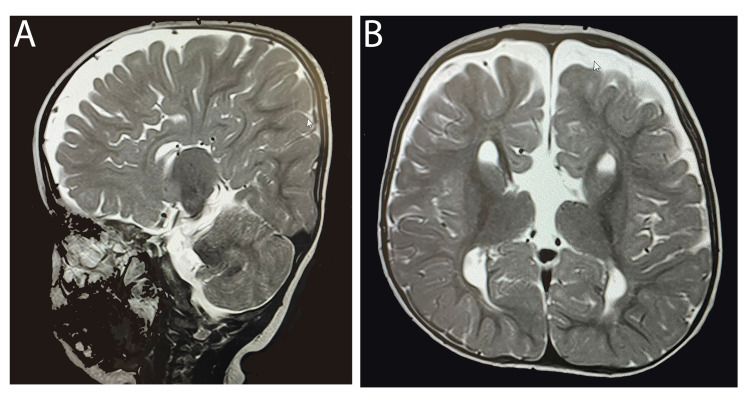
MRI of Case 1: (A) sagittal section (B) coronal section showing complete corpus callosum agenesis

A 14-month-old female child with a history of global developmental delay, dysmorphic features (hypertelorism, proptosis, ptosis, micrognathia), and amblyopia was referred to our hospital, King Abdulaziz University Hospital, Riyadh, Kingdom of Saudi Arabia, due to delayed speech and language development. She was born to first-degree consanguineous parents and was diagnosed as having bilateral severe to profound SNHL and fitted with bilateral hearing aids at the age of nine months. However, no clear benefits were detected from using hearing aids on follow-ups. Magnetic resonance imaging (MRI) revealed complete corpus callosum agenesis (Figure 1). In addition, whole-exome sequencing revealed an *LRP2* gene mutation, confirming the diagnosis of DBS. At the age of 18 months, her IQ test score was 75. 

At the age of 24 months, she was accepted by the hospital CI committee for bilateral sequential cochlear implantation. At 26 months of age, she received a right CI (SYNCHRONY with Form 24 electrode array, MED-EL Medical Electronics, Innsbruck, Austria) with no postoperative complications, and complete insertion of the electrode array was achieved. Two weeks later, the device was switched on. One year after implantation, aided audiometry revealed borderline normal to mild hearing range, whereas speech detection threshold (SDT) was 30 dB HL, and the IQ test score was 81. 

At the age of 46 months, the speech therapist reported overall fair performance. In June 2021, speech and language assessment revealed a LittlEARS® Auditory Questionnaire score of 35/35. This assessment tool is used to reflect the development of auditory behavior in children with CI [10]. In addition, an integrated scale of development tool was used to assess the progress in the six key areas of development, relative to hearing age and chronological age [11]. While at the time of evaluation her hearing age was 40 months, her scores were as follows: Audition: 19-24 months, Receptive language: 31-39 months, Expressive language: 19-24 months, Cognitive 43-48 months, Speech: 31-36 months. Categories of Auditory Performance (CAP) score was 5, which means that she could understand common phrases without lip reading [12]. In addition, her speech intelligibility rating (SIR) was 3, which indicated that her connected speech was intelligible to a listener who concentrates and lip-reads within a known context [13]. She attended a mainstream kindergarten where she spent 100% of the time in the mainstream classroom. Of note, she did not use sign language at any time as she effectively communicates verbally.

Case 2

A male child was born in July 2015, full-term, to first-cousin parents. He was the cousin of Case 1 who was referred to our outpatient clinic as a known case of DBS for developmental, speech, and language delay. Genetic analysis confirmed the diagnosis of DBS as a homozygous missense mutation of the *LRP2* gene was detected. The hearing assessment revealed bilateral severe to profound SNHL. Bilateral hearing aids were fitted; however, his aided threshold after six months was moderately severe to severe hearing level range. At the age of 28 months, his IQ score was 84. Computed tomography (CT) and MRI showed bilateral normal cochlea and cochlear nerves. 

**Figure 2:**
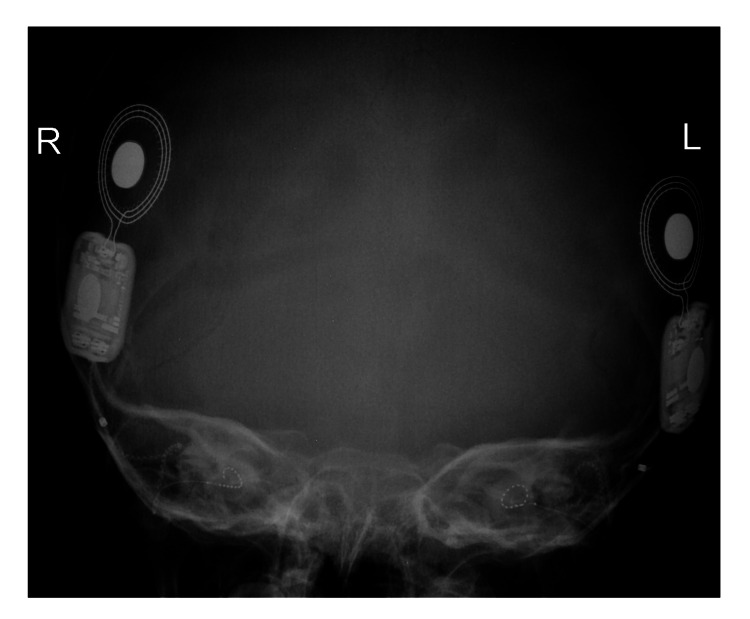
Postoperative x-ray temporal bone of Case 2 showing complete insertion of the cochlear implant electrode array at both ears

The patient was determined to be a candidate for bilateral implantation by the CI committee. At the age of three years and five months, bilateral simultaneous cochlear implantation (HiRes™ Ultra #D Cochlear Implant with right HiFocus™ SlimJ electrode and left HiFocus™ Mid-Scala electrode, Advanced Bionics AG, California, United States) was performed. The postoperative x-ray showed that the CI arrays were fully inserted inside the cochlea (Figure 2). Switch-on was done the next day after surgery and two Naida CI Q70 sound processors (Advanced Bionics AG, California, United States) were given. 

Three months after the switch-on, his parents reported adequate responses at home as the patient started to imitate words and vocalize. One month later, an aided assessment was tried using narrow-band noise and the average threshold was about 40 dB HL with SDT was 40 dB HL. The patient could discriminate three out of the six Ling sounds (a, i, u). One year after implantation, aided conditioned play audiometry revealed bilateral mild hearing range, the speech recognition threshold (SRT) for the right ear was 25 dB HL and for the left ear was 30 dB HL. The data logging was 10 hours/day. At the age of six years (his hearing age was about 31 months), the speech therapist conducted a speech and language assessment, which revealed a LittlEARS® score of 35/35 [10]. Upon using the integrated scale of development tracking [11], the performance of this patient was as follows: Audition: 31-36 months, Receptive language: 37-42 months, Expressive language: 31-46 months, Cognitive 43-48 months, Speech: 31-36 months. His CAP score was 7, which indicated his ability to use a telephone with a familiar talker [12]. Moreover, his SIR score was 4, which means that his speech is intelligible to a listener who has little experience of a deaf person’s speech [13]. He attended a mainstream kindergarten for the past two years where he spent 100% of the time in the mainstream classroom. Moreover, he did not use sign language at any time as he attained age-appropriate spoken language, which helps his effective verbal communication.

## Discussion

In this report, we studied the cases of two cousins with DBS, born from consanguineous marriages. Both patients were found to have global delayed development together with severe to profound SNHL. They did not benefit from using hearing aids, and both were accepted as candidates for bilateral cochlear implantation. Two years after the implantation, they reported improvement of hearing and speech perception, which helped them to attend mainstream schools, and they had not developed sign language. This is consistent with previous reports that showed improvement in verbal communication within two years of receiving implants [9,14]. 

The difference in the scores of CAP, SIR, and the integrated scale of development between the two cases can be explained by having different levels of preoperative IQ scores, the number of received implants, the presence or absence of corpus callosum agenesis, and being associated with different types of disabilities such as visual impairment [8]. In general, early recognition and diagnosis of DBS syndrome can improve the patients’ outcomes through guiding variable treatment interventions. Early audiological assessment for DBS patients should be done to diagnose any hearing impairment as early as possible, to maximize the beneficial outcome of the cochlear implantation. Furthermore, as parents of those patients are carriers of the mutated *LPR2* gene, prenatal genetic counseling is crucial, especially with consanguineous marriage.

## Conclusions

Our results showed that cochlear implantation could be considered as an approach for the management of DBS patients who suffer from bilateral SNHL. CI helped the two cases to restore hearing and to start to acquire language. The present data of the two reported cases provide new insights into the audiological management of DBS as a rare clinical disorder. Early detection and providing multidisciplinary care for those patients could help to provide them a better quality of life.
